# Eight-hour medication-assisted treatment waiver training for opioid use disorder: integration into medical school curriculum

**DOI:** 10.1080/10872981.2020.1847755

**Published:** 2020-11-23

**Authors:** Irvin C. Lien, Randell Seaton, Aaron Szpytman, Jody Chou, Victoria Webber, Eva Waineo, DianeL. Levine

**Affiliations:** a Department of Internal Medicine, Wayne State University School of Medicine, Detroit, MI, USA; b Department of Internal Medicine, Kaiser Permanente Oakland Medical Center, Oakland, CA, USA; c Department of Psychiatry and Behavioral Neurosciences, Detroit Medical Center, Detroit, MI, USA; d Department of Internal Medicine, Detroit Medical Center, Detroit, MI, USA

**Keywords:** Medical school curriculum development, opioid use disorder, medication-assisted treatment, student training, buprenorphine, MAT waiver

## Abstract

**Background**: The opioid epidemic is a growing problem in the USA. Use of medication-assisted treatment (MAT) has been effective in treating patients with opioid use disorders (OUD) and maintaining sobriety; however, there is a significant shortage of physicians formally trained in MAT.

**Objective**: Wayne State University School of Medicine integrated the 8-hour MAT waiver training into its Internal Medicine clerkship curriculum. The objectives of integrating this into the curriculum were to (1) introduce opioid use education during students’ Internal Medicine clerkship and (2) assess whether the curriculum prepares students to feel more comfortable evaluating and treating patients with OUD.

**Design**: MAT training specifically for medical students was provided free online by the Providers Clinical Support System (PCSS). All students on the Internal Medicine clerkship were required to complete the training. A 7-question pre-survey and post-survey assessed students’ comfort in evaluating and treating OUD. Significant changes were assessed with a paired McNemar Bowker Test.

**Results**: Medical students (n = 141) completed the pre-survey and post-survey. After the MAT training, students’ perspective of their clinical knowledge about OUD, familiarity with MAT, and likelihood to utilize MAT for their patients significantly differed, with increased proportions of medical students in agreement across 6 of 7 pre-post survey items (*p* <.0001).

**Conclusions**: Online MAT waiver training is a low-cost (free) way to introduce MAT education into the undergraduate clinical curriculum. Upon completing of the training, medical students self-reported improvements in their knowledge and attitudes about OUD and the different treatment options. Our hope is that MAT waiver training will allow for graduation of medical students who are ready to care for patients with OUD during residency and as practitioners upon completion of their residency.

## Introduction

Opioid use disorder (OUD) has rapidly become one of the most important health issues in the USA (US). In 2017, opioid overdose was reported to be the number one cause of accidental deaths (one in 96 persons) [1] and was responsible for approximately 47,600 deaths[2]. Despite decreased prescription of opioids, now at its lowest since 2006 [3], opioid overdoses continue to increase largely due to the advent of highly potent synthetic opioids such as fentanyl and carfentanil[4]. Use of medication-assisted treatment (MAT), including methadone and buprenorphine, can help patients with an OUD decrease their illicit drug use [5]. Training in MAT involves completion of an 8-hour curriculum in the evaluation and treatment of patients with OUD [6]. Unfortunately, there are not enough health-care providers in the US who are trained in MAT [7].

One proposed solution for relieving the MAT provider shortage is to introduce waiver training at the undergraduate level [8]. The American Association of Medical Colleges (AAMC) has encouraged medical schools to incorporate more education and assessment focused on OUD. An article published by the AAMC in January 2018 reported that 87% of the medical schools have self-reportedly addressed all the four major domains recognized to be important in an institute’s opioid use disorder curricula: (1) the nature of pain; (2) pain assessment and management; (3) management of pain, including substance use disorders and opioid overdose; and (4) context of pain and substance use disorders. The article noted three frequently reported challenges medical schools have faced in implementing these programs: faculty and resident development, time, and assessment [9]. MAT waiver training may be a potential avenue, as a well-developed and time-efficient method, to supplement a medical school’s OUD curriculum and address the challenges noted in this AAMC article.

Medical schools are beginning to introduce MAT training into their curriculum. A few medical schools were able to receive grants to incorporate MAT training so their medical students would complete the waiver training by graduation [10]. Without grant funding available, our school, like many others, needed to identify a way to incorporate MAT training into the curriculum. Utilization of this zero-cost 8-hour online MAT waiver training provided by the Providers Clinical Support System [6] allowed our school to deliver both training and assessment to all 300 students during their third year of medical school. We decided to place this training in the Internal Medicine clerkship when students are likely to see patients with OUD and have the opportunity to use this training. Undergraduate waiver training may result in more MAT-trained providers. The cost of this training, however, may be an obstacle to implementing this solution. Free online training may be a way past the obstacle, but its efficacy/quality is uncertain. We proposed to implement this form of training and assess its quality and impact through student self-assessment.

## Materials and methods

All third-year medical students on their eight-week Internal Medicine clerkship at the Wayne State University School of Medicine were required to complete the 8-hour MAT waiver training. In exchange for completing the training, they were granted an additional day off to study for their NBME Internal Medicine shelf examination.

The Providers Clinical Support System (PCSS) provides a free online version of the 8-hour MAT waiver training for anyone, including healthcare workers and medical students. Initially, the 8-hour MAT waiver training was accomplished through a 4.25-hour webinar offered twice a month, accompanied by a 3.75-hour online self-paced module. An additional option was provided by the PCSS to complete the entire 8-hour training as self-paced online modules [7]. Students could complete the training at any point during the clerkship. Upon completion, a certificate is provided and can be utilized as the 8-hour training portion of the practitioner waiver to prescribe buprenorphine under the Drug Addiction Treatment Act of 2000 (DATA 2000) [11].

Students were asked to complete a pre-survey, the 8-hour training, followed by a post-survey to assess the short-term impact on students’ knowledge and attitudes about opioid use disorders. The survey was initially piloted on a small sample of medical students to determine understandability and usability, then was revised accordingly. The survey (Table 1) consists of seven survey questions: three questions were related to clinical knowledge, two questions were related to attitudes towards OUD, one question was related to education on caring for patients with OUD, one question was related to their perspective on the utility of buprenorphine/naloxone in their future practice, and a question about their intended specialty. A 5-point Likert scale (1-strongly disagree to 5-strongly agree) was used to rate responses. Pre- and post-survey responses were paired for analysis using a McNemar Bowker Test with a significance level set at *p* < 0.05. A two-tailed Mann–Whitney U Test was used to identify the correlation between responses to question #1 and the students’ stated intended specialty.

**Table 1.** Pre-survey and post-survey results compiled from all medical students (*n* = 141) who completed the medication-assisted treatment waiver training

		Pre-MAT waiver training	Post-MAT waiver training	
	Survey question	*n* (%)	*n* (%)	*P* value
*Knowledge*				
3	I am knowledgeable about the opioid epidemic.			<.0001
	Agree^a^	76 (54)	121 (86)	
	Neutral	54 (38)	19 (13)	
	Disagree^b^	11 (8)	1 (1)	
4	I can identify a patient with opioid addiction and ask appropriate questions.			<.0001
	Agree	64 (45)	112 (80)	
	Neutral	53 (38)	27 (19)	
	Disagree	24 (17)	2 (1)	
5	I am familiar with buprenorphine/naloxone in medication-assisted treatment.			<.0001
	Agree	51 (36)	129 (91)	
	Neutral	49 (35)	11 (8)	
	Disagree	41 (29)	1 (1)	
*Skills*				
2	Based on my experience so far, the medical school and/or clinical rotations have prepared me to handle patients with opioid addiction.			*<*.0001
	Agree	50 (36)	83 (59)	
	Neutral	58 (41)	39 (28)	
	Disagree	33 (23)	19 (13)	
6	I feel comfortable managing a patient with opioid addiction.			<.0001
	Agree	21 (15)	91 (65)	
	Neutral	51 (36)	43 (30)	
	Disagree	69 (49)	7 (5)	
*Attitudes*				
1	The training would be beneficial to complete for my intended future specialty.			0.5808
	Agree	103 (73)	110 (78)	
	Neutral	34 (24)	27 (19)	
	Disagree	4 (3)	4 (3)	
*Future plans*				
7	I plan to prescribe buprenorphine/naloxone once I start practicing medicine.			<.0001
	Agree	49 (35)	88 (62)	
	Neutral	73 (52)	45 (32)	
	Disagree	19 (13)	8 (6)	

^a^Agree = Strongly agree + agree.

^b^Disagree = Strongly disagree + disagree

McNemar Bowker test was used to assess significant change.

The study was reviewed by the Wayne State University Human Investigation Committee and was found to not meet the definition of Human Participant research. Thus, Investigative Review Board approval was not required.

## Results

Between January 2019 and February 2020, 369 students completed the IM clerkship. Of those, 262 completed the pre-survey and 160 completed the post-survey; 141 students (38.2%) completed both. Participants who did not complete both the pre-survey and post-survey or had duplicate survey submissions were excluded from the analysis.

Statistically significant changes were found in students’ self-assessments of their knowledge, skills, and career future plans regarding OUD and MAT (questions 2–7) (Table 1). The largest change was the increase in the number of students who agreed or strongly agreed to the statement ‘I am familiar with buprenorphine/naloxone in medication-assisted treatment’ which increased from 36% (*n* = 51) in the pre-survey to 91% (*n* = 129) in the post-survey (Table 1). The smallest change was for the statement, ‘The training would be beneficial to complete for my intended future specialty’ which increased from 73% (n = 103) in the pre-survey to 78% (n = 110) in the post-survey (Table 1). Survey responses significantly differed (p < 0.0001) except for question #1: The training would be beneficial to complete for my intended future specialty (p = 0.5808).

Forty-one students (29%) intended to pursue primary care specialties (family medicine, internal medicine, or pediatrics) while 66 students (47%) intended to pursue non-primary care specialties. The remaining 34 students (24%) were unsure of their intended specialty. When comparing intended specialty differences for survey question #1 (The training would be beneficial to complete for my intended future specialty), only those declaring for psychiatry had a significant difference in perceptions that training would be useful for their specialty (p = 0.0172) (Table 2).

**Table 2.** Comparison of students in different intended specialties for question #1 ‘The training would be beneficial to complete for my intended future specialty.’

Specialty	*n*	Presurvey Likert mean of specialty	Presurvey Likert mean of all others	*p*-alue
Primary care				
Internal Medicine	29	4.28	4.04	0.2664
Pediatrics	7	3.43	4.13	0.1007
Family medicine	6	4.5	4.07	0.2677
Others				
Emergency medicine	23	4.35	4.04	0.1374
Surgery	18	3.89	4.12	0.6154
Psychiatry	7	4.86	4.05	0.0172
OB/GYN	5	4.20	4.09	0.9924
PM&R	3	4.67	4.08	*
Radiology	3	3.00	4.1	*
Ophthalmology	2	4.50	4.09	*
Anesthesia	1	4.00	4.09	*
Neurology	1	3.00	4.1	*
Radiology	1	3.00	4.1	*
Dermatology	1	3.00	4.1	*
Unsure	34	3.88	4.16	0.0516

^a^Sample size too small to conduct Mann–Whitney *U* test.

## Discussion

Our results indicate that after completing the 8-hour MAT training during the Internal Medicine clerkship, medical students felt improved competence and confidence regarding OUD and MAT, as well as increased intention towards using MAT in their future practice. The zero-cost PCSS online MAT training was a convenient and simple method of supplementing OUD education in the medical school curriculum that our students thought was helpful.

The only area that did not show a significant pre-survey to post-survey difference was students’ perception that the training would be beneficial for their intended specialty. This score was the highest on the pre-survey leaving little room for improvement. However, by further dissecting this question by specialty, significant pre-survey to post-survey changes were noted for psychiatry-bound students. It may be that medical students perceive MAT to be in the province of psychiatry, especially given that the bulk of substance use disorder didactics at our medical school are presented during the psychiatry curriculum. In addition, most students may already be familiar with MAT and the role their intended specialty had in treating OUD.

One of the primary limitations of this study is the absence of questions addressing negative attitudes towards MAT, OUD, and OUD patients, which has been identified as a primary cause for the lack of MAT providers [12]. Further studies inquiring about common misconceptions about MAT and OUD in medical students provide an opportunity to identify if undergraduate MAT training changes these misconceptions. At the beginning of the academic year, students were required to watch a scheduled 4.25-hour live webinar followed by a 3.75-hour online module, which only occurred twice a month. Informal verbal feedback from a few students was negative as they had difficulty carving out specific time in their schedule for the webinar. Later in the academic year, PCSS offered a new training consistent of entirely self-paced online modules. While all students received the PCSS training, this change in the PCSS-provided MAT training program delivery may have altered the individual student’s training experience and could have affected results. The restrictions and time commitment to complete the MAT wavier training may also explain why only 38.2% (141/369) of our students completed both the pre-survey and post-survey, causing a nonresponse bias. Additionally, our surveys only measured subjective self-assessment regarding OUD and did not contain objective evaluation in the content of learning. Future studies with objective questions regarding knowledge gained as well as long-term studies that measure the extended impact of this curriculum would be beneficial to further evaluate the impact of undergraduate MAT training. A study tracking the student group after residency training could determine if MAT training during medical school increases the number of physicians who obtain waivers and prescribe MAT.

## Conclusion

Zero-cost online MAT training was introduced into an Internal Medicine clerkship curriculum and was perceived by students to significantly improve their competence and confidence with MAT, OUD, and OUD patients. Other medical schools may adopt the MAT training into their third year as a simple, convenient, and free way to integrate OUD education into the undergraduate clinical curriculum.

## Data Availability

Raw data have been published on Figshare on the following website. https://doi.org/10.6084/m9.figshare.12142068
